# Influence of milk feeding levels and calf housing on subsequent performance of Holstein heifers

**DOI:** 10.3168/jdsc.2021-0077

**Published:** 2021-06-11

**Authors:** M.E. Prado, J. Wilkerson, L.G. Schneider, P.D. Krawczel

**Affiliations:** 1 Department of Animal Science, University of Tennessee, Knoxville 37996; 2 Department of Biosystems Engineering and Soil Sciences, University of Tennessee, Knoxville 37996; 3 Department of Agricultural Sciences, Faculty of Agricultural and Forestry, University of Helsinki, 00014, Helsinki, Finland; 4 Research Centre for Animal Welfare, Department of Production Animal Medicine, Faculty of Veterinary Medicine, University of Helsinki, 00014, Helsinki, Finland; 5 Helsinki One Health, University of Helsinki, 00014, Helsinki, Finland

## Abstract

The purpose of this study was to investigate the effects of increased milk feeding levels or social housing during the preweaning stage of Holstein dairy heifers on subsequent weight gain, health, and reproductive parameters over time. A total of 210 heifers were grouped by calving date in groups of 10 and randomly assigned to one of 3 treatments: low-milk individual housing (LMI; 4 L of milk/d), high-milk individual housing (HMI; 8 L of milk/d), or low-milk social housing (LMS; 4 L of milk/d). Data collected included passive transfer status, disease events, weight gain at different time periods, age at first calving, calving intervals, and milk yield over the first 3 lactations. Passive transfer was successful in 189 calves, and, overall, disease events were low in this herd. The average daily gain for heifers in the HMI group (0.79 ± 0.05 kg/d) was significantly higher (*F*_2, 9_ = 4.76) during the preweaning treatment period compared with the other groups (LMI: 0.59 ± 0.05 kg/d; LMS: 0.68 ± 0.04 kg/d). Although treatment groups did not differ with respect to age at first calving, the LMS and HMI heifers calved (22 and 15 d, respectively) earlier than the LMI heifers. Milk yield over 3 lactations was similar among the treatment groups. This suggests that both nutrition and social dynamics can influence performance of preweaned calves. Further investigation is needed to understand the mechanisms driving increased weight gain in socially housed calves.

## Highlights

• Increased milk allocation increased weight gain during the preweaning phase.
• Social housing tended to reduce age at first calving and increase weight gain during preweaning phase.
• Social housing or increased milk feeding did not negatively affect health.

## Graphical Abstract

**Figure:**
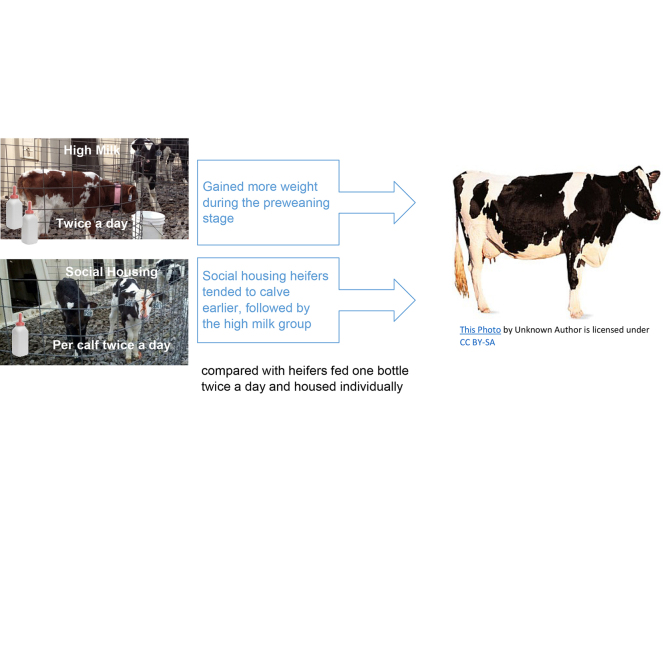
**Summary:** The purpose of this study was to investigate the effects of increased milk feeding levels (4 vs. 8 L) or housing (paired or individual) during the preweaning stage on subsequent weight gain, health, and reproductive parameters over time. Calves receiving a higher amount of milk gained more weight than calves receiving the standard amount. Furthermore, calves that were housed in pairs also gained more weight than traditionally housed and fed calves. This suggests that both nutrition and social dynamics can influence performance of preweaned calves. Further investigation is needed to understand the mechanisms driving increased weight gain in socially housed calves.

Most dairy operations separate calves from their dam within 24 h of birth (60% before 12 h of birth in the United States); 70% of these calves are placed in individual housing and then are routinely fed limited quantities of milk (4 to 6 L/d; USDA, 2016). Individual housing has been the standard of care for dairy calves primarily based on early studies of calf health. Earlier research suggested that increased morbidity and mortality occurred in group-housed calves compared with individually housed calves (Warnick et al., 1977). Individual housing was thought to reduce the risk for horizontal transmission of enteric and respiratory infections from calf to calf. Limit-feeding milk became the standard as a means to promote solid feed intake, but growth rates are reduced compared with calves provided higher planes of nutrition (Flower and Weary, 2001). However, these approaches to calf management are being re-evaluated.

There is growing evidence that early-life experience has long-term effects on the productivity of dairy cattle. Much of this has focused on the feeding strategy used during the milk feeding phase. Calves fed increased milk volumes exhibited more natural behavior compared with calves fed traditional milk amounts (Appleby et al., 2001; Jasper and Weary, 2002). There is also evidence of increased growth rates (Drackley, 2008) and greater long-term productivity (Soberon et al., 2012). Furthermore, early-life management can influence the general development of overall feeding behaviors that may have long-term implications (Miller-Cushon and DeVries, 2016). These findings highlight the potential for long-term effects on behavior, growth rates, and productivity; however, nutrition is only a portion of calf management.

The practice of individual housing has also been rethought, and there is evidence that it might be problematic for a variety of reasons. First, relative to socially housed calves, those raised individually are less prepared for their eventual transition to the social housing environment, common of lactating cows, as they are more timid than socially housed calves (Jensen and Larsen, 2014) and ranked lower within the social hierarchy (Veissier et al., 1994). Socially isolated calves were also less able to cope with novelty, as evidenced by increased heart rates when introduced to unfamiliar calves (Jensen et al., 1997) and more hesitancy to try new foods (Costa et al., 2014). In the short term, individually raised calves were less adaptive to new housing environments (De Paula Vieira et al., 2012) and engaged in a greater number of general fear behaviors (Horvath and Miller-Cushon, 2018) immediately after weaning. Over the long term, evidence is very limited, but a review by Costa et al. (2016) suggested that social housing (raising calves with extended contact with their dam or foster cow) may reduce fear and increase maternal behaviors.

Despite the evidence for positive benefits of social housing in the early stages of a calf's life or detrimental effects of social isolation, approximately 70% of dairy calves are still housed individually (USDA, 2016). The lack of adoption of social housing is due to the continued concern that the risk of disease is greater than the benefits gained from social housing as well as the lack of clear indicators of the economic consequences of poor management of preweaned calves (Sumner and von Keyserlingk, 2018). Collectively, this suggests that data on long-term growth or milk yield performance are critical to determine the effects of early housing on the productive life of dairy cattle. We hypothesized that heifers receiving a higher milk allocation would gain more weight during the preweaning period and would produce more milk in the first lactation than calves receiving restricted milk allocation or calves that are socially housed. Therefore, the objective of this study was to test the effects of housing and milk feeding levels on health and performance of dairy heifers over several lactations.

The Institutional Animal Care and Use Committee at the University of Tennessee approved the experiments performed in this study (protocol no. 2112-0715). A 1,000-cow closed commercial dairy farm was used. All lactating cows were milked 3 times per day, housed in either compost-bedded pack pens or freestalls, and fed a TMR. Upon calving, 210 heifer calves were enrolled in the study, and male calves were sold shortly after birth in accordance with the farm's management strategy. Data were recorded for female calves enrolled, including ID number, birth date, sire, dam, birth weight, number of calves born (single or multiple), and a dichotomous calving ease score (normal or assisted). Calves enrolled in the study were born between October 2012 and March 2013.

Calves were separated from their respective dam within 5 h of birth. At that time, navels were disinfected with diluted iodine and each calf was tube-fed 3.8 L of colostrum. An additional 3.8 L was given approximately 12 h later. Calves were administered a polyvalent intranasal vaccine against bovine respiratory syncytial virus, infectious bovine rhinotracheitis, and parainfluenza 3 virus and were given a commercial bolus containing antibodies against *Escherichia coli* K99+ and bovine coronavirus. Between 7 and 21 d of age, a *Clostridium* 7-way vaccine was given, and between 34 and 49 d of age, modified live respiratory viruses (infectious bovine rhinotracheitis, parainfluenza 3, bovine respiratory syncytial virus, and bovine viral diarrhea) and a bacterin against *Mannheimia haemolytica* and *Pasteurella multocida* were administered.

Following birth, calves were housed in individual pens in a nursery for approximately 2 wk. They were then grouped by calving date into groups of 10 to maintain homogeneity and moved to housing in hutches (Calf-Tel Pro Hutch), where treatments were initiated. Each group of 10 calves (experimental unit) was randomly assigned to one of 3 treatments: low-milk individual housing (**LMI**; 4 L of milk/d; n = 50), high-milk individual housing (**HMI**; 8 L of milk/d; n = 60), or low-milk social (pair) housing (**LMS**; 4 L of milk/d; n = 100). Due to restrictions placed by the producer, a fourth treatment group receiving 8 L of milk/d and social housing could not be added. The average age at the time of movement from the individual pens to the hutches (preweaning treatment period) was 19.1 ± 5.21 d for LMS (range: 8–25 d), 12.9 ± 5.43 d for LMI (range: 6–25 d), and 19.7 ± 7.77 d for HMI. Calves in the HMI treatment were transitioned to this volume over 7 d to allow for adjustment to the higher volume. For practical reasons, the 8 L of milk was provided in 2 bottles per feeding. This volume represented twice the amount of the standard milk diet and approximated 20% of BW in milk allocation for the average dairy calf. Regardless of treatment, all hutches were located on a gravel pad and included a fenced-in yard at the front of each hutch. For LMS calves, 2 adjacent hutches were grouped by fencing to allow access to an outside area that was double the single hutch area. The producer maintained the usual routine for calf management except for the level of milk feeding and housing arrangement. At the end of the study period, calves were moved into groups of 10 heifers each to a transition shelter (d 61–102). The transition shelter consisted of 4 pens (3.7 m × 7.3 m) with access to automatic waterers and a feed trough. The pens were bedded with straw.

Diet consisted of farm waste milk, free-choice water, and a calf starter ration. Milk was fed in standard bottles twice per day during the preweaning treatment period. Afterward, calves (d 61–102) were transitioned to group pens (10 calves/pen) and were fed commercial waste milk by mob feeder for approximately 2 wk followed by weaning. Milk for this group was purchased from a commercial processing plant and consisted of a mix of chocolate milk, buttermilk, and other milk by-products. Preweaning calves were offered creep pellets and water ad libitum. Following the group pen period, heifers were moved to a pasture with access to grass hay and commercial feed.

Five milliliters of whole blood was collected from each calf during the first week of life by jugular venipuncture into standard red-top tubes for determining passive transfer of antibodies. Blood was allowed to clot, and the serum was separated by centrifugation (20°C for 10 min at 900 × *g*), placed in cryovials, and stored at −80°C until ready for analysis. Passive transfer was assessed by single radial immunodiffusion (Triple J Farms). Passive transfer was considered adequate if IgG concentration was ≥1,000 mg/dL. Failure of passive transfer (**FPT**) was defined as IgG concentration <1,000 mg/dL (Dawes et al., 2002).

Before the start of the study, the calf manager and other farm employees received standard training and appropriate case definitions for each disease of interest to reduce the possibility of subjective assessments. Morbidities were recorded by the calf manager based on a calf health scoring chart previously described by Poulsen and McGuirk (2009). Disease events of interest included respiratory disease, diarrhea, navel infection, or joint infections. In addition to recording the event, farm workers recorded the treatment administered, frequency of treatment, and the outcome (death or survival through weaning and 80 d postweaning observation). Disease monitoring occurred by visual observation of each calf at the time of feeding for the following parameters: depression, decreased appetite, nasal or ocular discharge, ear position, cough, and fecal consistency. When deemed necessary, the joints were evaluated for heat or swelling, and abnormal gait and navels were assessed for swelling or discharge. Treatment decisions were made using the expertise of the herd veterinarian and routine farm treatment protocols. A veterinarian involved in this study verified the calf manager's diagnoses using the calf health scoring charts and the farm veterinarian's treatment records. The farm was visited at least once a week, during which time calf enrollment sheets and morbidity scoring charts were collected. All calves were individually monitored from birth to approximately 100 d of age for signs of respiratory disease, diarrhea, and any other signs of morbidity.

Respiratory disease was reported in any calf exhibiting clinical signs corresponding to a score of 5 or more (Poulsen and McGuirk, 2009); diarrhea was reported in calves voiding abnormal feces classified as loose or watery; and joints or navel infection were reported in calves displaying clinical signs of swelling (heat, swelling, discharge, and abnormal gait) in those anatomical locations.

Individual BW was collected using a portable scale at birth from a subset of the calves and at entry into the calf hutches (preweaning treatment period), at exit from the calf hutches, and monthly in group pens from all of the calves. The ADG was determined during the preweaning and group pen periods separately by dividing the weight gained in each period by the number of days spent in each period.

Once heifers reached breeding age or weight (~12 mo/340 kg), PGF_2α_ was administered to synchronize their heat cycle. This protocol was repeated 2 wk later if no heat was observed. Heifers were bred by AI using sexed semen. A Double Ovsynch program was used for lactating cows starting around d 42 postpartum. The farm data base was accessed to compile age at first calving, calving interval between lactations 1 and 2, DIM, average milk per day, and total milk yield in 305 d for the first 3 lactations. Cows that died or were removed from the herd before completing 305 d of lactation were not included in the analysis.

As mentioned above, sets of calves born at similar times were treated as a group and randomly assigned to the same treatment; therefore, calf group was considered the experimental unit. Within each calf group, individual calves were the sampling units. To determine the effects of milk allocation and housing in calves, a completely randomized study with sampling was implemented. Separate mixed-model ANOVA models were developed using the GLIMMIX procedure of SAS (SAS Institute Inc.). The fixed effect of treatment and the random effect of calf birth group nested within treatment were included in all models. Normality of residuals, homogeneity of variance, and diagnostic plots were assessed to ensure that ANOVA assumptions were met using the UNIVARIATE procedure. When assessing treatment effects on growth parameters, the covariate of birth weight was included as a fixed effect. If treatment differences were observed (*P* < 0.05), contrasts were used to test specific treatment differences. Least squares means and standard errors of the means are reported, as well as *F*-statistics and degrees of freedom. To test whether there was an association between treatment and remaining in the herd until the first lactation, a chi-squared analysis was performed, and the chi-squared statistic and *P*-value are reported. Statistical significance was determined at α = 0.05, and trends were declared at *P* < 0.09.

Sample size for this study was calculated using the UnifyPow SAS macro. Using an unbalanced completely randomized design with 5 reps per treatment group and 10 calves (control or high milk) or 20 calves (social housing) in each group, we determined that a sample size of 160 or 196 total calves would be required to have 80 or 90% power, respectively, to detect an effect of treatment on ADG. Parameters used included mean ADG of 0.6, 0.7, and 0.8 kg/d for LMI, LMS, and HMI groups, respectively, SD = 0.25, and α = 0.05.

There were no differences in birth weights (*F*_2, 9_ = 0.69, *P* = 0.53) between treatment groups or while calves were in the nursery (*F*_2, 9_ = 0.92, *P* = 0.43) before the beginning of the study (d 17; Table 1). After adjusting for the covariate of calf birth weight (*P* < 0.001), calves in the HMI group tended to have increased BW (*F*_2, 9_ = 3.99, *P* = 0.06) at 61 d of age, with LMS calves as an intermediate during the preweaning treatment period. During the postweaning period when calves were moved to the group pens, there were no differences in final BW, indicating that LMS and LMI calves experienced compensatory growth and reached weights comparable with those of the HMI calves. Alternatively, HMI heifers may have had reduced weight gain in the postweaning period due to reduced milk availability, lower calf starter intake and, thus, lower rumen development (Sweeney et al., 2010; Mirzaei et al., 2018). Growth rates (ADG) in the preweaning treatment period differed among treatments (*F*_2, 9_ = 4.76, *P* = 0.04), with HMI calves having the highest ADG and LMS calves serving as the intermediate and not differing from HMI or LMI.

**Table 1:** Growth parameters (mean ± SE) of young dairy calves during the pretreatment, treatment, and posttreatment periods

Item	Treatment^1^			*P*-value
	LMI	HMI	LMS	
Calves, no.	39	39	80	
BW, kg				
Birth^2^	40.2 ± 0.7^a^	39.1 ± 0.7^a^	39.3 ± 0.5^a^	0.53
Nursery^2^ (d 17)	40.5 ± 0.9^a^	42.2 ± 0.9^a^	41.2 ± 0.9^a^	0.43
Preweaning^3^, ^4^ (d 61)	66.1 ± 3.2^a^	78.5 ± 3.2^a^	68.9 ± 3.2^a^	0.057
Group pen^4^, ^5^ (d 102)	122.7 ± 5.2^a^	125.5 ± 5.2^a^	120.4 ± 5.0^a^	0.78
ADG,^3^ kg/d				
Preweaning^3^, ^4^ (d 17–61)	0.59 ± 0.05^b^	0.79 ± 0.05^a^	0.68 ± 0.04^ab^	0.04
Group pen^4^, ^5^ (d 61–102)	1.20 ± 0.07^a^	1.20 ± 0.07^a^	1.17 ± 0.07^a^	0.93

a–c Means within a row with different superscripts differ (α < 0.05).

1 Treatments were instituted during the preweaning treatment period (d 17–61) and consisted of low-milk individual housing (LMI; 4 L of milk/d; n = 50), high-milk individual housing (HMI; 8 L of milk/d; n = 60), or low-milk social (pair) housing (LMS; 4 L of milk/d; n = 100).

2 Data collected during the pretreatment period.

3 Data collected during the treatment period.

4 Least squares means adjusted for birth weight covariate (*P* < 0.02).

5 Data collected during the posttreatment period.

Overall, this result is in line with what was expected from previous work suggesting that increased provision of milk results in greater BW and ADG (Drackley, 2008; Borderas et al., 2009). In terms of ADG, the observed gain in the current study aligned very well with the values predicted by Drackley (2008). It was predicted that a conventional nutritional approach to calf feeding (i.e., limited milk and ad libitum access to grain) would lead to growth rates between 0.5 and 0.6 kg/d (Drackley, 2008); our observed ADG of 0.59 kg/d is within that range. Similarly, accelerated growth programs (i.e., increased milk with ad libitum access to grain) would generate growth rates between 0.6 and 0.8 kg/d (Drackley, 2008); our calves provided increased access to milk achieved growth of 0.79 kg/d. Interestingly, our LMS calves grew at 0.68 kg/d, which is consistent with the performance that Drackley (2008) predicted with moderate increases in milk allocation. This suggests that LMS can facilitate increases in growth rate similar to those in the accelerated programs without the additional costs of providing greater volumes of milk.

Although these calves were not evaluated in a way that provides the means to draw conclusions on the mechanisms for this growth rate, there are a few potential explanations. We subjectively observed that LMS calves consumed more grain than HMI and LMI calves. However, the amount of grain consumed was not determined in this experiment. Limiting milk intake leads to increased grain consumption (Borderas et al., 2009). However, Jensen et al. (2015) observed increased grain intake only in pair-housed calves receiving an increased allocation of milk. Still, there is evidence of social housing decreasing the fear of novel feeds. Calves raised in complex social environments consumed greater amounts of novel foods and approached novel food options more readily (Costa et al., 2014). Therefore, it is possible that LMS calves consumed more concentrate in the current study relative to individually housed calves. The LMS calves were also anecdotally occupying the same hutch throughout the day. As cold weather increases the maintenance requirements of calves (Drackley, 2008), there is also the potential that sharing space decreased the maintenance requirements of these calves. Further studies are merited to establish the aspects of social housing that might promote improved ADG.

Passive transfer occurred in 189 of 210 calves as determined by radial immunodiffusion. There were 13, 4, and 4 calves with FPT in the LMS, HMI, and LMI groups, respectively.

All calves developed diarrhea approximately 10 d after transitioning into hutches (preweaning treatment period) and were treated by the farm manager per farm protocols. Diarrhea was self-limiting, and calves maintained a good appetite during the duration of this event.

The level of clinical disease for calves during the treatment period was low. Two out of 210 calves were diagnosed with respiratory disease; both calves were in the LMI group. One calf was confirmed by the diagnostic laboratory as having bovine respiratory syncytial virus. The second calf was suspected of having *Mycoplasma* sp.; however, her diagnostic tests were inconclusive. Both calves were treated by the farm manager per farm protocols and fully recovered. Furthermore, 33 calves (6 traditional, 11 high milk, and 16 social housing) were observed to be “off” by the farm manager and were treated with antibiotics per farm protocols. None of the calves had specific clinical signs, and they did not meet the criteria for a clinical score >5 using the Wisconsin scoring chart (Poulsen and McGuirk, 2009). Signs noted by the farm manager were moving slower than or not being as active as the rest of the calves and normal appetite. Four (1 LMS, 1 LMI, 2 HMI) of the 33 calves treated with antibiotics had FPT.

The volume of colostrum fed at the first feeding (3.8 L) and the total fed within the first 24 h (7.6 L) were greater than that fed on 58 and 51% of US dairy operations, respectively (USDA, 2016). Only 10% of calves were categorized as having FPT of immunity, which is consistent with the mean response on farms with more than 500 cows and slightly better than the typical farm in the eastern region of the United States (Shivley et al., 2018). The low morbidity rates (0.95% of calves were diagnosed with respiratory disease) were less than half the mean rate reported in the United States (Gorden and Plummer, 2010). Collectively, the low FPT and low morbidity rates suggest that colostrum management on the farm was effective, which is likely a critical component of successful social housing.

Treatment groups did not differ with respect to age at first calving (*P* = 0.30); however, there were numerical differences worth noting. Heifers in the LMS group calved approximately 22 d earlier (662.7 ± 9 d) than heifers in the LMI group (684.2 ± 10.1 d) posttreatment. The mean calving age of HMI heifers was 15 d earlier (669.2 ± 9.2 d) than that of LMI heifers and 7 d later than that of LMS heifers. Calving intervals for lactations 2 and 3 were not different (*P* < 0.29).

Age at first calving was within the range of the optimal recommended age of 22 to 24 mo (Hoffman, 1997). No statistical differences were seen in age at first calving; however, it was interesting that heifers in the LMS group calved numerically earlier than the other 2 groups. As mentioned above, social housing with limited milk allocation may lead to increased grain consumption and growth rate and thus result in attaining breeding weight earlier.

Out of 210 heifers from the original preweaning study, 194 reached first lactation. Thirteen heifers died (2 LMI, 3 HMI, and 2 LMS) or were sold (6 LMS) for various reasons. Milk yield at 305 d was similar among the treatment groups (*P* < 0.46). There were no significant effects on milk yield at 305 d during the second and third lactations (Table 2). Previous studies demonstrated that proper nutrition during the preweaning period affects early development and future productivity (Davis Rincker et al., 2011). In addition, BW before calving was associated with total milk yield in the first lactation (Heinrichs and Heinrichs, 2011; Chester-Jones et al., 2017). It was beyond the scope of this study to monitor the heifers' weight gain posttreatment. Although we did not see a significant difference between groups in the first lactation or the subsequent 2 lactations, a plausible explanation may lie in the limitation on how randomization of the treatment groups took place. In this study, the farm management practices limited our ability to randomly allocate calves individually to each treatment group. Calves were grouped by birth age and moved together to hutches for practical reasons. These groups were assigned a treatment group and constituted a block. When the analysis was performed considering each calf a unit (except for the LMS calves, which were considered one unit together), the data produced significant differences in weight gain and milk yield.

**Table 2:** Total milk yield (kg; (mean ± SE) by lactation for cows in the experimental treatments

Lactation	Treatment^1^			*P-*value
	LMI	HMI	LMS	
1	10,537 ± 511	10,729 ± 452	9,976 ± 390	0.44
2	10,964 ± 778	10,943 ± 682	9,907 ± 526	0.39
3	8,358 ± 1,247	7,645 ± 1,107	6,875 ± 855	0.57

1 Treatments consisted of low-milk individual housing (LMI; 4 L of milk/d; n = 50), high-milk individual housing (HMI; 8 L of milk/d; n = 60), or low-milk social (pair) housing (LMS; 4 L of milk/d; n = 100).

Heifer calves receiving a higher amount of milk gained more weight than calves receiving the standard amount during the preweaning period. Similarly, LMS calves tended to gain more weight than LMI calves. Furthermore, LMS heifers calved earlier than heifers in the HMI and LMI groups. This suggests that both nutrition and social dynamics can influence performance of preweaned calves. No differences were found between treatment groups in weight gain, health, and milk yield for 3 lactations; this may be in part because calves were blocked by age in groups of 10 and randomly assigned a treatment group, limiting the number of units. Further studies with a larger sample size are needed to determine whether higher milk diets and social housing significantly affect weight gain during the preweaning period, health, calving age, and lactation.
